# The impact on primary care of a large waterborne campylobacter outbreak in Norway: a controlled observational study

**DOI:** 10.1080/02813432.2023.2299116

**Published:** 2024-02-07

**Authors:** A. Iversen, G. Rortveit, K. A. Wensaas, C. O. Gulla

**Affiliations:** a Department of Global Public Health and Primary Care, University of Bergen, Bergen, Norway; b Chief Medical Officer’s Staff, Askøy, Norway; c Research Unit for General Practice, NORCE Norwegian Research Centre, Bergen, Norway; d National Centre for Emergency Primary Health Care, NORCE Norwegian Research Centre, Bergen, Norway

**Keywords:** Campylobacter infection, gastroenteritis, primary health care, out-of-hours services, general practice, disease outbreaks, *Campylobacter jejuni*

## Abstract

**Objective:**

Document the impact of an outbreak of gastroenteritis on local primary health care services, compared to a control period.

**Design:**

Controlled observational study with data from the outbreak and a control period. Data obtained from electronic medical records (EMR) of general practitioners (GPs) and the out-of-hours (OOH) service. Telephone data from the OOH service’s telephone records.

**Setting:**

Campylobacteriosis outbreak in Askøy municipality, Norway in 2019. Over 2000 individuals were infected.

**Subjects:**

Patients in contact with GPs and the OOH service during the outbreak and a control period.

**Main outcome measures:**

Patient contacts with GPs and the OOH service during the outbreak and a control period.

**Results:**

There was a 36% increase in contacts during the outbreak compared to the control period (4798 vs. 3528), with the OOH service handling 78% of outbreak-related contacts. Telephone advice was the dominant method for managing the increase in contacts to primary care, both in OOH services and daytime general practice (OR 3.73 CI: [3.24–4.28]). Children aged 0–4 years had increased use of primary care during the outbreak (OR 1.51 CI: [1.28–1.78]). GPs referred 25% and OOH services referred 75% of 70 hospitalized cases.

**Conclusion:**

The OOH service handled most of the patients during the outbreak, with support from daytime general practice. The outbreak caused a shift towards telephone advice as a means of providing care. Young children significantly increased their use of primary care during the outbreak.

## Background

Infection with *Campylobacter spp.*, known as campylobacteriosis, is a common cause of gastroenteritis outbreaks in Nordic countries, and *Campylobacter jejuni* is often the involved subspecies [1]. In Norway, outbreaks are often a result of contaminated drinking water, while preparation and consumption of poultry and barbeque meals are other frequent routes of transmission [2]. Norway has around 3000 reported cases of campylobacteriosis every year, with a peak during summer [3]. Most cases are not part of a recognized outbreak and around 50% of the cases are imported. Worldwide, *Campylobacter spp.* are estimated to be the cause of 400 to 500 million cases of gastroenteritis annually [4].

Campylobacteriosis is a self-limiting infection that seldom requires antimicrobial therapy [5] and mortality is low, even for patients with bacteraemia [6]. Infection is more common in children and young adults. Clinical features are indistinguishable from gastroenteritis due to other bacteria. Abdominal cramps, abdominal pain, fever, and diarrhoea, with or without bloody stools, are typical symptoms in adults [7,8]. Fever and vomiting are also common, and bloody stools occur more frequently in children [9].

In Norway, all municipalities are required by law to have a daytime general practice with general practitioners (GP), an out-of-hours (OOH) service, and chief medical officer(s) with the capability to manage outbreaks. The OOH service is organized by the municipalities and generally staffed by local GPs, in addition to nurses and other health personnel.

In June 2019 there was a large outbreak of campylobacteriosis in Askøy in Norway [10]. At the time there were 25 GPs working office hours and an OOH service with one GP, serving a population of 29,500. The outbreak was discovered June 6 after an increased number of patients with symptoms of gastroenteritis sought assistance at the local OOH service.

More than 2000 individuals are assumed to have been infected during this outbreak, and 70 patients were hospitalized [11]. Two deaths were related to the outbreak, an infant and an elderly person both with comorbidity [12]. The outbreak sparked intense national media attention.

The outbreak originated from an elevated basin in the water supply system, serving about 12,000 local residents and the regional centre with commercial and administrative offices.

An initial boil water advisory was sent *via* SMS to a limited municipal area on June 6 at 18:02, followed by a press release to the media at 19:46, and a subsequent advisory to the entire municipality at 19:48 [13].

To manage the outbreak, the number of on-duty GPs was doubled from June 6 to 11, and extra nurses and an additional telephone station were activated.

The elevated basin closed on June 7 [14]. *C. jejuni* was identified in faecal samples from a hospitalized patient June 7, and on June 11 *C. jejuni* was found in water samples from the basin and connected sites. The boil water advisory lasted until 18 July [11].

The aim of this study was to document the impact of the outbreak on local primary health care services by comparing with a control period.

## Methods

In this controlled observational study in Askøy, real-time data were collected for two periods: the outbreak period (June 3–16) and the control period (May 13–26, 2019), chosen for their proximity and similar holiday timings (Whit Monday on June 10 and Norwegian National Day on May 17).

Data from electronic medical records (EMR) of all 25 GPs and the OOH service in Askøy were gathered. This included all physical and telephone contacts, using Microsoft Excel 16.0 and a custom program for semi-automated data collection and validation, with collection of sex and age being fully automated.

Our case definition for gastroenteritis was the use of the ICPC-2-diagnosis D73 ‘Gastroenteritis presumed infection’ or the recorded presence of two or more of the following symptoms: diarrhoea, vomit, abdominal pain, nausea, fever, and intestinal gas pain.

For each contact in the two periods, we collected contact reason (gastroenteritis within the case definition, outbreak concern or other), contact type (consultation, telephone advice with health personnel, doctor’s visit, ambulance or other), date and place of contact, sex of the patient, and year of birth.

The value “other” for contact reasons was defined as anything but gastroenteritis or outbreak concern, while the value “other” for contact types includes contacts concerning billing, opening hours, prescription renewal, etc.

For case identification, we included symptoms and diagnoses registered in GPs/OOH service’s schedules and information from follow-up contacts, if available in the periods we examined.

Age was calculated by subtracting the year of birth from 2019. With no patients born before 1920 in our dataset, there was no risk for century calculation errors.

Telephone data from the OOH service’s electronic telephone system was collected in a separate dataset using the telephone system’s report module.

### Statistical analyses

STATA 18.0 (StataCorp, Texas, USA) was used for statistical analyses. We used Chi square to test differences between outbreak and control periods.

We calculated odds ratios (OR) with 95% confidence intervals (CI) with the outbreak period as exposure and the control period as reference for variables (Tables 1 and 2). OR was not calculated when there were no comparable contacts during both periods. The difference in mean pick-up-time for incoming calls was analysed using a *t*-test.

**Table 1.** Summarized characteristics for all contacts with daytime general practice and out-of-hours service in the outbreak period and the control period.

	All	Outbreak	Control		
	*N* = 8326	*n* = 4798 (58%)	*n* = 3528 (42%)	OR [95% CI]^a^	Chi Square^b^
Sex					
Female	4698	2667 (56%)	2031 (58%)	0.92 [0.84–1.01]	
Male	3628	2131 (44%)	1497 (42%)	1.08 [0.99–1.18]	0.071
Age range (yrs.)^c^					
0–4	679	452 (9%)	227 (6%)	1.51 [1.28–1.78]	
5–14	656	380 (8%)	276 (8%)	1.01 [0.86–1.19]	
15–24	982	542 (11%)	440 (13%)	0.89 [0.78–1.02]	
25–34	1039	617 (13%)	422 (12%)	1.09 [0.95–1.24]	
35–44	1046	622(13%)	424 (12%)	1.09 [0.96–1.24]	
45–54	1277	748 (16%)	529 (15%)	1.05 [0.93–1.18]	
55–64	886	461 (10%)	425 (12%)	0.78 [0.67–0.89]	
65–74	923	526 (11%)	397 (11%)	0.97 [0.85–1.11]	
75–84	590	323 (7%)	267 (8%)	0.88 [0.75–1.04]	
≥ 85	248	127 (3%)	121 (3%)	0.77 [0.59–0.99]	< 0.000
Contact reasons					
Gastroenteritis	1316	1288 (27%)	28 (1%)	45.87 [31.46–69.47]	
Outbreak concern	141	141 (3%)	NA^d^	NA	
Other	6869	3369 (70%)	3500 (99%)	0.02 [0.01–0.03]	< 0.000
Contact types					
Consultation	5393	2830 (59%)	2563 (73%)	0.54 [0.49–0.60]	
Telephone advice	1486	1197 (25%)	289 (8%)	3.73 [3.24–4.28]	
Other^e^	1338	717 (15%)	621 (18%)	0.82 [0.73–0.93]	
Doctor’s visit	77	37 (1%)	40 (1%)	0.68 [0.43–1.09]	
Ambulance^f^	32	17 (0%)	15 (0%)	0.83 [0.39–1.79]	< 0.000

^a^Outbreak as exposure.

^b^Chi square p-value per group (sex, age, contact reason and contact type).

^c^Maximum age 99 yrs. First group spans 5 yrs.

^d^Not applicable.

^e^Billing, opening hours, prescription renewal, etc.

^f^Assessment or physician advice to ambulance regarding a patient.

**Table 2.** Summarized characteristics of contacts with daytime general practice and out-of-hours service during the outbreak and control period.

	Daytime general practice				Out-of-hours service			
	Outbreak	Control		Chi square^b^	Outbreak	Control		Chi square
	*n* = 2957	*n* = 2802	OR [95% CI]^a^	*p-*value	*n* = 1841	*n* = 726	OR [95% CI]	*p*-value
Sex								
Female	1684 (57%)	1631 (58%)	0.95 [0.85–1.06]		983 (53%)	400 (55%)	2.99 [6.61–3.41]	
Male	1273 (43%)	1171 (42%)	1.05 [0.95–1.17]	.334	858 (47%)	326 (45%)	3.10 [2.69–3.58]	.436
Age range (yrs.)^c^								
0–4	129 (4%)	112 (4%)	1.10 [0.84–1.43]		323 (18%)	115 (16%)	2.86 [2.29–3.60]	
5–14	150 (5%)	190 (7%)	0.73 [0.58–0.92]		230 (13%)	86 (12%)	2.66 [2.06–3.47]	
15–24	341 (12%)	347 (12%)	0.92 [0.78–1.08]		201 (11%)	93 (13%)	2.12 [1.64–2.76]	
25–34	378 (13%)	337 (12%)	1.07 [0.91–1.26]		239 (13%)	85 (12%)	2.81 [2.17–3.66]	
35–44	397 (13%)	362 (13%)	1.05 [0.89–1.22]		225 (12%)	62 (9%)	3.64 [2.72–4.93]	
45–54	500 (17%)	444 (16%)	1.08 [0.94–1.25]		248 (14%)	85 (12%)	2.93 [2.26–3.81]	
55–64	322 (11%)	351 (13%)	0.85 [0.72–1.00]		139 (8%)	74 (10%)	1.82 [1.35–2.46]	
65–74	410 (14%)	348 (12%)	1.14 [0.97–1.33]		116 (6%)	49 (7%)	2.29 [1.62–3.29]	
75–84	236 (8%)	224 (8%)	0.99 [0.82–1.21]		87 (5%)	43 (6%)	1.94 [1.33–2.88]	
≥ 85	94 (3%)	87 (3%)	1.02 [0.75–1.39]	.067	33 (2%)	34 (5%)	0.92 [0.55–1.53]	<.000
Contact reasons								
Gastroenteritis	301 (10%)	18 (1%)	17.53 [10.86–30.06]		987 (53%)	10 (1%)	139.88 [75.43–293.22]	
Outbreak concern	12 (0%)	NA^d^	NA		129 (7%)	NA	NA	
Other	2 644(90%)	2 784 (99%)	0.55 [0.32–0.88]	<.000	725 (40%)	716 (99%)	0.94 [0.84–1.07]	<.000
Contact types								
Consultation	2 228 (75%)	2 253 (80%)	0.74 [0.66–0.85]		602 (33%)	310 (43%)	2.05 [1.76–2.39]	
Telephone advice	87 (3%)	21 (1%)	4.01 [2.45–6.83]		1 110 (60%)	268 (37%)	5.68 [4.89–6.60]	
Other^e^	635 (22%)	518 (19%)	1.21 [1.06–1.38]		82 (5%)	103 (14%)	0.75 [0.55–1.01]	
Doctor’s visit	7 (0%)	10 (0%)	0.66 [0.21–1.93]		30 (2%)	30 (4%)	0.95 [0.56–1.63]	
Ambulance^f^	0 (0%)	0 (0%)	NA	<.000	17 (1%)	15 (2%)	1.07 [0.50–2.31]	<.000

^a^Outbreak as exposure.

^b^Chi square per group (sex, age, contact reason and contact type).

^c^Maximum age 99 yrs. First group spans 5 yrs.

^d^Not applicable.

^e^Billing, opening hours, prescription renewal, etc.

^f^Assessment or physician advice to ambulance regarding a patient.

## Results

### Patient contacts with general practice and OOH services

**Figure 1.**
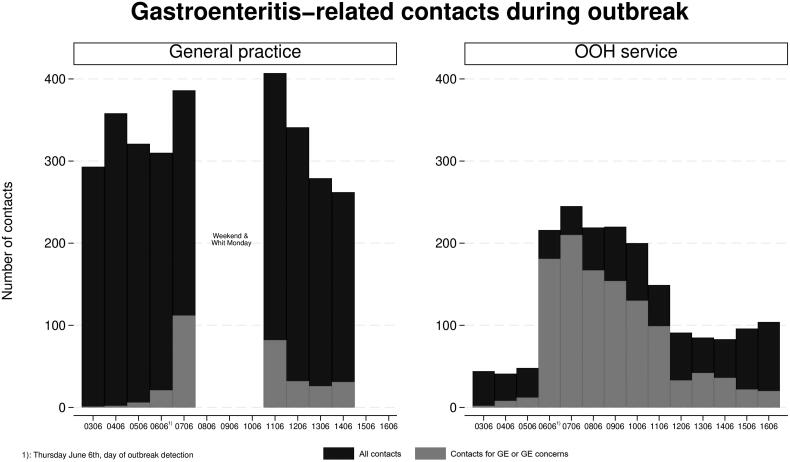
Combined number of gastroenteritis and gastroenteritis concern contacts (grey) shown together with number of all contacts (black), during the outbreak for general practice and out-of-hours service.

We found a 36% increase in contacts during the outbreak compared to the control period (4798 vs. 3528) (Table 1). Gastroenteritis or outbreak concern accounted for 1429 contacts (30% of all) in primary care during the outbreak; 1116 (78%) at OOH services and 313 (22%) in daytime general practice. Outbreak-related contacts peaked in the first two days and then gradually declined (Figure 1).

Contacts by telephone increased almost fourfold during the outbreak period compared to the control period (1197 vs. 289). Due to this increase, the proportion of physical consultations decreased, although the absolute number of such consultations increased slightly (2830 vs. 2563), resulting in an OR of 0.54 CI: [0.49–0.60]. The same was the situation for contacts categorized as “other” (717 vs. 621; OR 0.82 CI: [0.73–0.92]).

Patients aged 0-4 years used primary health care more in the outbreak period compared to the control period (OR 1.51 CI: [1.28–1.78]) and had the most cases during the outbreak (*n* = 193). Age groups 55-64 and ≥ 85 had a relative reduction in primary health care use. For all other age groups there were no changes (Table 1).

From June 7 to June 11, 75% of contacts to the OOH services were outbreak related, at daytime general practice this proportion was 29% (supplementary material Table 1). Of all contacts related to the outbreak, 81% (1156) were during these five days.

**Figure 2.**
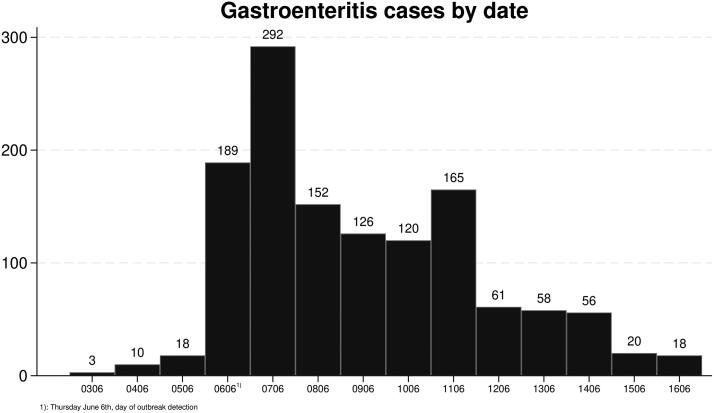
Gastroenteritis cases during the outbreak period, shown by contact date with OOH service or daytime general practice.

We found 31 contacts for gastroenteritis in the three days prior to the outbreak being detected on June 6 (Figure 2), 22 in daytime general practice and 9 at the OOH service.

### OOH service

The number of contacts increased 154% (1841 vs. 726) during the outbreak compared to the control period.

The number of contacts due to gastroenteritis was much larger during the outbreak compared to the control period (987 vs. 10). Another 129 contacts were caused by outbreak concern, for a total of 1116 outbreak related contacts with OOH services, representing 60% of all contacts to the OOH services during the outbreak period (Table 2).

For all age groups, there were more contacts during the outbreak compared to the control period, apart from patients ≥ 85 years (Table 2). Adults aged 35-44 contacted the OOH service more in the outbreak (225 vs. 62) and the age 0-4 group had the largest absolute increase (323 vs. 115) between the two periods.

Use of telephone advice was much more frequent in the outbreak period compared to the control period (1110 vs. 268), as was the case for consultations (602 vs. 310) (Table 2).

The number of incoming calls increased substantially during the outbreak period (2689 vs. 926). In the control period, 90% of the calls were answered, compared to 72% in the outbreak period. Mean call duration was similar in the two periods (238 vs. 229 sec), but mean pick-up-time increased from 23 sec in the control period to 70 sec during the outbreak (Supplementary material Table 2).

**Figure 3.**
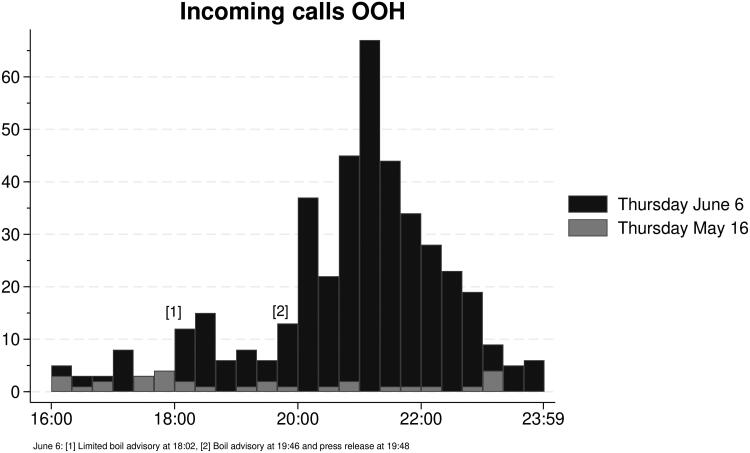
Incoming calls on Thursday June 6, when the outbreak became publicly known, compared to Thursday May 16 (control period) at the out-of-hours service in Askøy municipality. Group width is fifteen minutes.

Telephone capacity was exceeded on the evening of June 6 (Supplementary material Figure 1). This coincided with the time for the two boil advisories and the first press release about the outbreak issued by local authorities (Figure 3).

### Daytime general practice

During the outbreak period there was a minor increase (6%) in the total number of contacts as compared to the control period (2957 vs. 2802), with no changes in sex or age distribution (Table 2).

Gastroenteritis was the cause of 301 contacts during the outbreak, compared to 18 contacts in the control period. Another 12 contacts were caused by outbreak concern, making the total number of outbreak related contacts with daytime general practice 313, 11% of all registered contacts during the outbreak.

Telephone advice was used more frequently during the outbreak (87 vs. 21), and the number of consultations was reduced (2228 vs. 2253). The use of other types of contact were unchanged (Table 2).

### Hospitalization and the gate keeper role of primary care

In our material, we identified 64 of the 70 known cases of gastroenteritis that were referred to hospital during this outbreak. GPs referred 16 (25%) and OOH services referred 48 (75%) of these cases. The referral rate was 5% for both GPs (16 of 301) and the OOH service (48 of 987).

## Discussion

### Principal findings

There was a 36% increase in contacts during the outbreak compared to the control period, and the OOH service handled 78% of outbreak-related contacts.

Gastroenteritis was the reason for 1288 contacts with primary care during the outbreak, compared to 28 cases of gastroenteritis during the control period. The increase in overall contacts during the outbreak (*n* = 1270) was almost exclusively due to gastroenteritis.

Telephone advice was the dominant method for managing the abrupt increase in contacts to primary care during the outbreak, both in OOH services and daytime general practice.

Children aged 0–4 years had an increased use of primary care during the outbreak. Even though this age group only spans five years, it also had the largest increase in number of contacts (323 vs. 115) between the two periods.

### Strengths and weaknesses

The OOH service is localized in the municipality’s centre, where the outbreak had its epicentre and 23,500 of the municipality’s 29,500 inhabitants lived. Our data includes all GP and OOH service contacts in the municipality during the outbreak and control periods, which significantly strengthens the ability to assess the gatekeeper role of primary care.

Given Askøy’s island geography, residents typically use local health services, making it less likely they sought care elsewhere. However, the missing six cases of known gastroenteritis-cases that were referred to the hospital, may have been referred from other sources than GPs or the OOH service in Askøy, or by direct hospital admittance *via* ambulance, bypassing the local primary health care services.

During the outbreak, two researchers were active in the municipality: AI in the chief medical officer’s staff and COG as a daytime GP and OOH doctor. Their involvement added contextual depth to the research but also raised the possibility of a biased presentation of their work and results. This was mitigated by real-time data analysis and the involvement of GR and KAW in result interpretation and presentation. The use of a semi-automated computer program with data validation rules for EMJ data collection also minimized registration errors.

There is a risk of misclassification of gastroenteritis cases. We may have missed some cases, due to the lack of symptoms described in patient journals. Ascertainment bias might also be present in the outbreak period, due to unintentional diligent interpretation of symptoms by medical staff.

The control period was selected to mimic the outbreak period: both periods have a public holiday connected to the weekend in the middle of the periods, and the periods are closely connected in time to reduce seasonal variations in patient health care seeking behaviour. However, it is still possible that the periods differ in properties we have not considered.

The surge in primary care contacts mainly occurred in the OOH service. This service expanded its hours and capacity, recruiting extra doctors from municipal GPs, impacting regular general practice activity. Additionally, the outbreak’s timing just before a weekend meant OOH was the sole primary care provider for three days at the outbreak’s onset. Had the outbreak started at a different time, it would likely have had a more significant impact on the utilization of daytime general practice.

### Findings in relation to other studies

General practice and OOH services in Norway are intertwined and the ability to adjust to increased demand of primary health services over longer periods have already been described [15]. Our study documents and evaluates the rapid adjustments made during the sudden spike in primary healthcare demand starting on June 6 and the subsequent days.

The disease burden of gastroenteritis is comparable to that of influenza-like illness [16] and is managed within the regular capacity of primary care. Consultations for gastroenteritis account for a larger proportion of the total workload in OOH services compared to daytime general practice [17]. During outbreaks of an infectious disease OOH services may increase their overall capacity [18], whereas daytime primary care may adapt by reducing consultations for other reasons [15]. This is in line with the findings in our study.

Diarrhoea is a leading cause of death amongst children under five years old [19] and acute gastroenteritis was the second most common non-traumatic cause of emergency hospitalization in children aged 1–5 years in a recent German study [20]. In the Askøy outbreak, one infant died with *C. jejuni*-gastroenteritis as a contributing cause of death. Young children are often brought to OOH services with gastroenteritis [21], and our findings reveal a significant rise in primary care contacts for young children during this outbreak, particularly in those under five years old.

Telephone advice is the recommended measure for OOH services in Norway for abdominal pain with vomiting and diarrhoea when the patient is not flaccid or exhausted [22]. Increased use of telephone advice, as we found in our data, eliminates the risk of pathogen transmission by attendance, and still enables evaluation of care providers’ and/or the patients’ assessment of the situation, thus reducing the need for consultations [15,23], while the risk of under-triage remains low [24].

The OOH service referred 75% of the hospitalized cases, while the remaining cases were referred from daytime general practice. Referral rates from OOH service for acute illness is known to be larger than from daytime general practice [25], and in our setting, this phenomenon is reinforced since the start of the outbreak was just before a weekend and Whit Monday, when daytime general practice was closed.

Little is known about the gatekeeper role of primary care during an outbreak of this scale. The referral rates of gastroenteritis we found, with 92% of hospitalized cases emanating from primary care, shows that the gate keeper role of primary can be preserved under an outbreak of this scale, even with a backdrop of an infant death and substantial media attention.

### Implications

Our study shows that at the start of an outbreak of gastroenteritis of this size, the capacity of the health care system is challenged. There was a rapid increase in the number of contacts to both daytime general practice and OOH services, but for several reasons this was most evident in the latter. Management of the situation calls for the services to be available for patients, at least by telephone, a proper triage system and sufficient capacity to assess patients at risk of more serious disease.

OOH services should have facilities, equipment and staff that allow for a rapid upscaling in the event of an outbreak. The role of daytime general practice should be integrated in such plans due to its potential to reorganize its services and manage more emergency cases, and as a source of support if the OOH service needs more resources.

Coordination of all primary care health services should be part of plans for infection and outbreak control, as there seems to be an unused potential for outbreak management in daytime general practice.

In Norway there is currently no available infrastructure that enables a rapid research response in primary care during an outbreak or an event of similar proportions. To enhance our understanding and examination of primary healthcare services, we believe that the establishment of reporting and automated, real-time monitoring systems is essential.

## Conclusions

During a large outbreak of gastroenteritis caused by *Campylobacter*, most cases were managed only in primary care. The major increase in contacts to primary health care were handled mostly by the local OOH service, with support from daytime general practice. The outbreak caused a shift towards the telephone being used as a means of triaging and providing care.

Children aged 0–4 years had the largest increase in the use of primary care during the outbreak.

The role as gatekeeper for specialist health services by primary care is evident in our study.

## Supplementary Material

Supplemental Material

Supplemental Material

## References

[1] Guzman-Herrador B, Carlander A, Ethelberg S, et al. Waterborne outbreaks in the nordic countries, 1998 to 2012. Eurosurveillance. 2015;20(24):21160. doi: 10.2807/1560-7917.ES2015.20.24.21160. 26111239

[2] Kapperud G, Espeland G, Wahl E, et al. Factors associated with increased and decreased risk of Campylobacter infection: a prospective case-control study in Norway. Am J Epidemiol. 2003;158(3):234–242. doi: 10.1093/aje/kwg139. 12882945

[3] Norwegian Surveillance System for Communicable Diseases. https://statistikk.fhi.no/msis/sykdomshendelser?etter=diagnose&fordeltPaa=aar&diagnose=301&maaned=1,2,3,4,5,6,7,8,9,10,11,12&diagramtype=soyle&tidsrom=2013,2018

[4] Quintel BK, Prongay K, Lewis AD, et al. Vaccine-mediated protection against Campylobacter-associated enteric disease. Sci Adv. 2020;6(26):eaba4511. doi: 10.1126/sciadv.aba4511. 32637610 PMC7314533

[5] Butzler JP. Campylobacter, from obscurity to celebrity. Clin Microbiol Infect. 2004;10(10):868–876. doi: 10.1111/j.1469-0691.2004.00983.x. 15373879

[6] O’Hara GA, Fitchett JRA, Klein JL. Campylobacter bacteremia in london: a 44-year single-center study. Diagn Microbiol Infect Dis. 2017;89(1):67–71. doi: 10.1016/j.diagmicrobio.2017.05.015. 28629878

[7] Yoo M, Chung SH, Park YS, et al. Clinical characteristics of Campylobacter enterocolitis in Korean adults: a retrospective study at a single center. Korean J Gastroenterol. 2020;75(4):188–197. doi: 10.4166/kjg.2020.75.4.188. 32326685

[8] White AE, Ciampa N, Chen Y, et al. Characteristics of Campylobacter and Salmonella infections and acute gastroenteritis in older adults in Australia, Canada, and the United States. Clin Infect Dis. 2019;69(9):1545–1552. doi: 10.1093/cid/ciy1142. 30602004 PMC6606397

[9] Puthucheary SD, Parasakthi N, Liew ST, et al. Campylobacter enteritis in children: clinical and laboratory findings in 137 cases. Singapore Med J. 1994;35(5):453–456. 7701360

[10] Emberland KE, Wensaas KA, Litleskare S, et al. Clinical features of gastroenteritis during a large waterborne Campylobacter outbreak in askoy, Norway. Infection. 2021;50(2):343–354. doi: 10.1007/s15010-021-01652-3. 34215942 PMC8942940

[11] Health NIoP. Utbrudd av Campylobacter, Askøy: Norwegian Institute of Public Health; 2019.

[12] Mortensen N, Jonasson SA, Lavesson IV, et al. Characteristics of hospitalized patients during a large waterborne outbreak of Campylobacter jejuni in Norway. PLOS One. 2021;16(3):e0248464. doi: 10.1371/journal.pone.0248464. 33755697 PMC7987138

[13] SINTEF. uavhengig gransking av hendelse ved Kleppe vannverk 2019. 2021.

[14] Hyllestad S, Iversen A, MacDonald E, et al. Large waterborne *Campylobacter* outbreak: use of multiple approaches to investigate contamination of the drinking water supply system, Norway, june 2019. Euro Surveill. 2020;25(35):2000011. 32885779 10.2807/1560-7917.ES.2020.25.35.2000011 PMC7472686

[15] Simonsen KA, Hunskaar S, Sandvik H, et al. Capacity and adaptations of general practice during an influenza pandemic. PLOS One. 2013;8(7):e69408. doi: 10.1371/journal.pone.0069408. 23874960 PMC3715475

[16] Schmutz C, Bless PJ, Mäusezahl D, et al. Acute gastroenteritis in primary care: a longitudinal study in the swiss sentinel surveillance network, sentinella. Infection. 2017;45(6):811–824., doi: 10.1007/s15010-017-1049-5. 28779435 PMC5696444

[17] Emberland KE, Wensaas KA, Litleskare S, et al. Consultations for gastroenteritis in general practice and out-of-hours services in Norway 2006-15. Fam Pract. 2019;36(5):614–620. doi: 10.1093/fampra/cmy133. 30689824 PMC6781938

[18] Ramerman L, Rijpkema C, Bos N, et al. The use of out-of-hours primary care during the first year of the COVID-19 pandemic. BMC Health Serv Res. 2022;22(1):679. doi: 10.1186/s12913-022-08096-x. 35597939 PMC9122805

[19] Collaborators G. Estimates of the global, regional, and national morbidity, mortality, and aetiologies of diarrhoea in 195 countries: a systematic analysis for the global burden of disease study 2016. Lancet Infect Dis. 2018;18(11):1211–1228. 30243583 10.1016/S1473-3099(18)30362-1 PMC6202444

[20] Posovszky C, Buderus S, Classen M, et al. Acute infectious gastroenteritis in infancy and childhood. Dtsch Arztebl Int. 2020;117(37):615–624. 33263539 10.3238/arztebl.2020.0615 PMC7805585

[21] Wolters PI, Holtman G, Fickweiler F, et al. Referral rates for children with acute gastroenteritis: a retrospective cohort study. BJGP Open. 2020;4(3):bjgpopen20X101053. doi: 10.3399/bjgpopen20X101053. PMC7465583 32694136

[22] National Centre for Emergency Primary Health Care NNRC. Legevaktindeks. 2022. https://legevaktindeks.no/.

[23] Morreel S, Philips H, Verhoeven V. Organisation and characteristics of out-of-hours primary care during a COVID-19 outbreak: a real-time observational study. PLOS One. 2020;15(8):e0237629. doi: 10.1371/journal.pone.0237629. 32790804 PMC7425859

[24] Gamst-Jensen H, Lippert FK, Egerod I. Under-triage in telephone consultation is related to non-normative symptom description and interpersonal communication: a mixed methods study. Scand J Trauma Resusc Emerg Med. 2017;25(1):52. doi: 10.1186/s13049-017-0390-0. 28506282 PMC5433057

[25] Blinkenberg J, Pahlavanyali S, Hetlevik O, et al. Correction to: general practitioners’ and out-of-hours doctors’ role as gatekeeper in emergency admissions to somatic hospitals in Norway: registry-based observational study. BMC Health Serv Res. 2020;20(1):876. doi: 10.1186/s12913-020-05590-y. 32938473 PMC7493318

