# Supplementary material for: The impact on primary care of a large waterborne campylobacter outbreak in Norway: a controlled observational study
Source: Scand J Prim Health Care. 2024 Feb 7;42(1):187–94. doi: 10.1080/02813432.2023.2299116 (PMC10851797; doi:10.1080/02813432.2023.2299116)
Supplement: Supplemental Material [file IPRI_A_2299116_SM9375.docx]

**Supplementary material table 1** Activity during the five most busy days of the outbreak, June 7 to June 11.

|  | | ***Total*** | ***OOH Service*** | **Daytime general practice** |
| --- | --- | --- | --- | --- |
|  | | *n= 2352* | *n= 1 249* | *n = 1 103* |
| Contact reasons | |  |  |  |
|  | Gastroenteritis | 1 044 (44%) | 839 (67%) | 205(19%) |
|  | Outbreak concern | 112 (5%) | 102 (8%) | 10 (10%) |
|  | Other | 1 196 (51%) | 308 (25%) | 888 (81%) |
|  | |  |  |  |
| Contact types | |  |  |  |
|  | Consultation | 1 165 (50%) | 356 (29%) | 809 (73%) |
|  | Telephone advice | 894 (38%) | 833 (67%) | 61 (6%) |
|  | Other^4)^ | 269 (11%) | 39 (3%) | 230 (21%) |
|  | Doctor’s visit | 16 (1%) | 13 (1%) | 3 (0 %) |
|  | Ambulance^5)^ | 8 (0%) | 8 (1%) | 0 (0 %) |

**Supplementary material table 2** Comparison of duration, pick-up and number of inbound telephone
calls at out-of-hours service in Askøy during the outbreak period and control period.

|  | | **Outbreak** |  | **Control** |  |
| --- | --- | --- | --- | --- | --- |
|  | | *n* |  | *n* | *OR [95% CI]* ^1)^ |
| Total Inbound calls | | 2 689 |  | 926 | NA^2)^ |
|  | Answered | 1 927 (72%) |  | 833 (90 %) | 0.28 [0.22-0.36] |
|  | Unanswered | 762 (28%) |  | 93 (10 %) | 3.54 [2.81-4.46] |
|  |  |  |  |  |  |
|  | |  |  |  |  |
|  | | *sec.* |  | *sec.* | p-value |
| Inbound calls^3)^ | |  |  |  |  |
|  | Avg. duration, 95 % CI^1)^ | 241 [233-249] |  | 228 [216-239] | 0.18 |
|  | Avg. pick-up-time, 95 % CI^1)^ | 70 [63-77] |  | 23 [21-25] | 0.0 |

1) Outbreak as exposure. 2) Not applicable 3) Calls shorter than 5 seconds and two calls > 1500 seconds not included.
