# Supplementary material for: The impact on primary care of a large waterborne campylobacter outbreak in Norway: a controlled observational study
Source: Scand J Prim Health Care. 2024 Feb 7;42(1):187–94. doi: 10.1080/02813432.2023.2299116 (PMC10851797; doi:10.1080/02813432.2023.2299116)

**Supplementary material figure 1**: Incoming calls to out of service during outbreak period, grouped by dropped and answered calls.


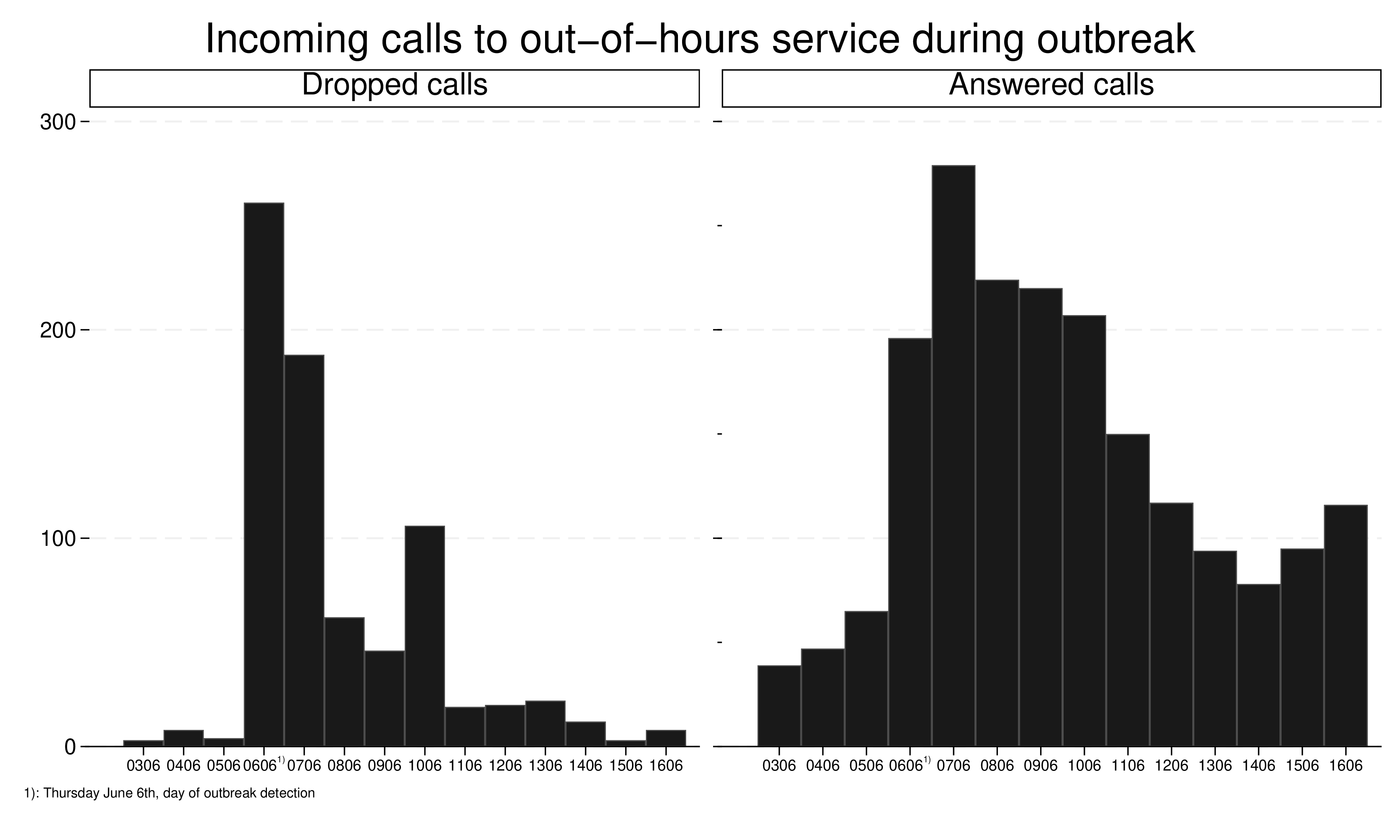

Supplement: Supplemental Material [file IPRI_A_2299116_SM9372.docx]
